# On‐Surface Synthesis of Unsaturated Hydrocarbon Chains through C−S Activation

**DOI:** 10.1002/chem.202200809

**Published:** 2022-07-11

**Authors:** Luca Giovanelli, Rémy Pawlak, Fatima Hussein, Oliver MacLean, Federico Rosei, Wentao Song, Corentin Pigot, Frédéric Dumur, Didier Gigmes, Younal Ksari, Federica Bondino, Elena Magnano, Ernst Meyer, Sylvain Clair

**Affiliations:** ^1^ Aix-Marseille Univ, CNRS, IM2NP Marseille France; ^2^ University of Basel Department of Physics Basel CH4056 Switzerland; ^3^ Key Laboratory of Functional Materials Physics and Chemistry of the Ministry of Education Jilin Normal University Changchun 130103 China; ^4^ Institut National de la Recherche Scientifique Varennes Québec J3X 1S2 Canada; ^5^ Aix-Marseille Univ, CNRS, ICR Marseille France; ^6^ IOM-CNR Laboratorio TASC AREA Science Park, Basovizza 34149 Trieste Italy; ^7^ Department of Physics University of Johannesburg PO Box 524 Auckland Park 2006 South Africa

**Keywords:** core-level photoemission spectroscopy, homocoupling, polyacetylene, radical coupling reaction, scanning probe microscopy, thiophene

## Abstract

We use an on‐surface synthesis approach to drive the homocoupling reaction of a simple dithiophenyl‐functionalized precursor on Cu(111). The C−S activation reaction is initiated at low annealing temperature and yields unsaturated hydrocarbon chains interconnected in a fully conjugated reticulated network. High‐resolution atomic force microscopy imaging reveals the opening of the thiophenyl rings and the presence of *trans*‐ and *cis*‐oligoacetylene chains as well as pentalene units. The chemical transformations were studied by C 1s and S 2p core level photoemission spectroscopy and supported by theoretical calculations. At higher annealing temperature, additional cyclization reactions take place, leading to the formation of small graphene flakes.

## Graphical abstract

**C−S activation** is used in a surface‐supported reaction to create a reticulated network of conjugated hydrocarbon chains on Cu(111). The reaction is initiated at low annealing temperature and the product is characterized by high‐resolution scanning probe microscopy and photoelectron spectroscopy supported by DFT calculations.

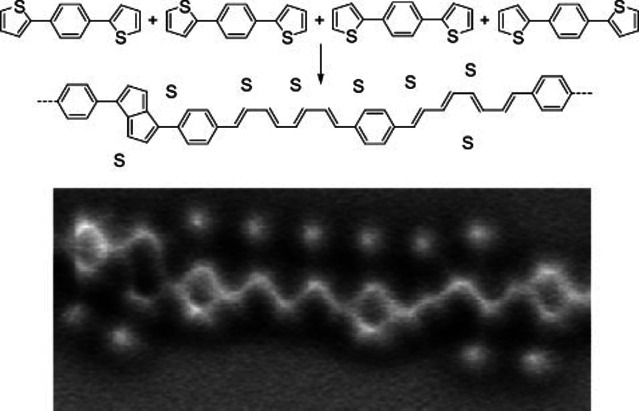

## Introduction

A variety of chemical reactions have been explored in on‐surface synthesis to create organic compounds by taking advantage of a solid surface acting as a confining template.^[1]^ Inspired by the Ullmann reaction, the C−C coupling between halogenated precursors was demonstrated^[1a]^ and now represents the most widely used approach. A few other mechanisms have also proven successful, most remarkably the direct C−H activation that is greatly facilitated by the supporting metal surface.^[2]^ While alternate reaction pathways have been scarcely proposed,^[3]^ it is of prime importance to investigate reaction mechanisms for on‐surface synthesis to gain better control and predictability over the reaction products and to position this emerging field as an efficient and versatile chemical synthesis approach.

In traditional solvent‐based chemistry the transition metal‐mediated activation of C−S bonds has been extensively investigated and sulfur‐based organic synthesis is emerging as an alternative to halogen‐based synthesis.^[4]^ Thiophenyl groups represent an attractive way of introducing sulfur in organic precursors. Thiophene derivatives could be successfully implemented in 3D covalent organic frameworks (COFs) for efficient gap tuning.^[5]^ On surfaces, a few covalent networks containing thiophene units have been obtained from Ullmann coupling on Ag(111)^[6]^ or Cu(110).^[7]^ Pure polythiophene chains were reported on Au(111) from dibrominated oligothiophenes.^[8]^ While quaterthiophene and heptathiophene molecules are stable at room temperature on Cu(111),^[9]^ in most cases, a degradation of thiophene derivatives is observed upon thermal activation on reactive surfaces^[10]^ like Cu(111)[^6a^, ^6c^, ^11^] or Ni(111).^[12]^ Thiophene degradation can be also obtained upon application of an electric field by the STM tip^[13]^ or by applying atomic hydrogen.^[14]^ Successful C−S bond activation has been shown to initiate intra‐ or inter‐molecular reaction on a surface.^[12b]^ In this latter work the thiophene units were fused with adjacent benzene rings, which probably limited the reactivity of the as‐released radicals and the possibility to extend the polymerization reaction.

**Figure 1:**
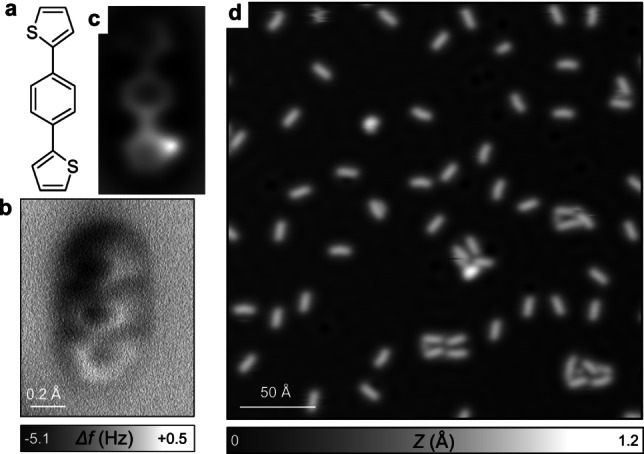
a) 1,4‐di(thiophen‐2‐yl)benzene (DTB) precursor. b) High‐resolution AFM image of a single DTB obtained with a CO‐functionalized tip and c) corresponding image simulation (DFT). d) STM image of isolated DTB molecules on Cu(111) acquired at 4.8 K.

We designed the precursor 1,4‐di(thiophen‐2‐yl)benzene (DTB, Figure 1a)^[15]^ as a model system to demonstrate on‐surface C−S bond activation as an efficient mechanism to perform C−C coupling at moderate annealing temperature. The thiophene unit in DTB is linked to the core benzene ring through a single C−C bond, a configuration that enables good flexibility compared to a fused thiophene ring.^[12b]^ Electropolymerization of this molecule^[16]^ and of similar threefold precursors^[17]^ has been reported to create materials with good conductivity or electrochromic properties. The polymeric system obtained in this work is fully conjugated and composed mainly of oligoacetylene segments separated by benzene rings. Low‐temperature atomic force microscopy (AFM) imaging using CO‐terminated tips combined with photoemission spectroscopy and density functional theory (DFT) reveals the structure of the extended polymeric chains, with a mixture of *cis*‐ and *trans*‐ polyene as well as pentalene, which are globally aligned along the substrate high‐symmetry directions. Our work thus positions C−S bond activation as an effective strategy in the on‐surface synthesis approach, delivering compounds with high potential in molecular electronics applications under mild conditions.

## Results and Discussion

DTB molecules were deposited on a Cu(111) surface kept at room temperature in a submonolayer regime. At room temperature the molecules are highly mobile and form a 2D gas phase. Upon cooling the substrate down to 4.8 K, the molecules become immobilized enabling the acquisition of stable scanning tunneling microscopy (STM) images (Figure 1d). DTB molecules exhibit very weak intermolecular interaction since no large self‐assembly can be observed. Figure 1(b) shows an AFM image using a CO‐terminated tip (see Methods in Supporting Information) of an isolated molecule that confirms its chemical structure. The molecules remain intact upon sublimation with the benzene and thiophene rings lying almost flat on the surface.[^9^, ^18^] The thiophene rings are slightly tilted and appear elongated by AFM and terminated by a bright protrusion or a depression corresponding to the sulfur atom, as confirmed by the simulated AFM image using DFT coordinates of the relaxed DTB structure on Cu(111) (Figure 1c and Supporting Information Figure S1). Several conformers coexist on the surface which differ according to the *syn*‐ or *anti*‐ orientation of the thiophene units with respect to the central phenyl (see Supporting Information Figure S1).

**Figure 2:**
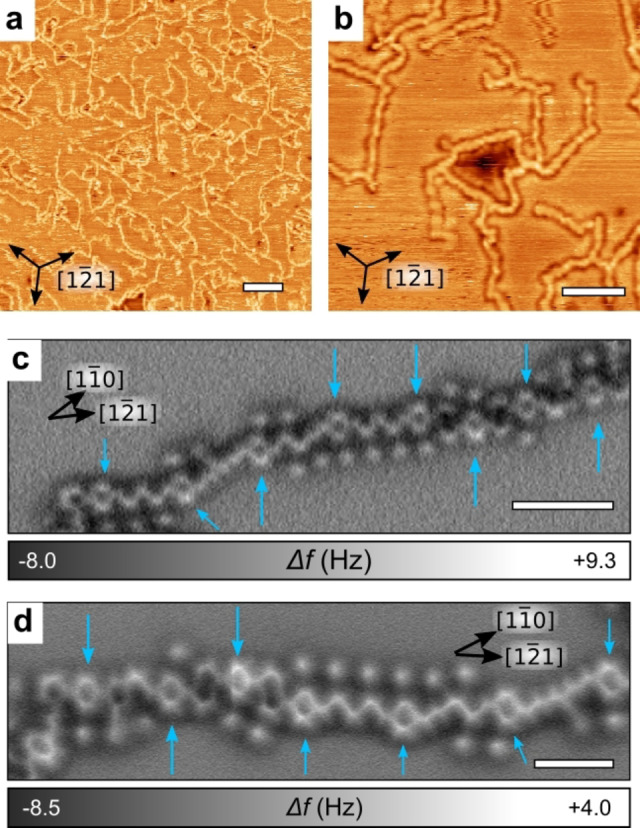
a, b) RT‐STM images revealing the on‐surface reaction of DTB on Cu(111) after annealing at a) 150 °C or b) 200 °C. c, d) High‐resolution AFM images of the polymeric chains obtained after annealing at 130 °C showing the preserved benzene rings (blue arrows) regularly distributed and linked by linear carbon chains. Scale bars: (a) 10 nm, (b) 4 nm, (c, d) 1 nm.

An annealing temperature of 150 °C was required to induce the full C−S activation and the opening of all thiophene rings, although partial reaction could already be observed after 80 °C annealing (see Figure S2). STM images acquired at room temperature (Figure 2a, b) show the formation of a polymeric network consisting of straight chains up to ∼20 nm long and with a tendency to align with the [1$\bar{2}1$ ] substrate directions. The chains are randomly interconnected, forming an extended 2D reticulated network. High‐resolution AFM images confirmed that the chains consist of the original benzene rings of DTB interconnected by different configurations of oligoacetylene chains (Figure 2c, d). The chains and benzene rings are resolved with a homogeneous contrast, indicating a perfectly flat adsorption configuration and suggesting that the sulfur atoms were fully removed from the polymer. The contrast of the carbon chains resembles the *trans‐* or *cis*‐polyacetylene chains that were obtained on Cu(110)^[19]^ and has the same periodicity (2.4 Å and 4.4 Å for the *trans*‐ and the *cis*‐chains, respectively). DFT calculations further indicate that the most favorable orientation for the *trans*‐chains is along the [$1\bar{1}0$ ]‐direction (see Supporting Information Figure S3a). The *trans*‐polyacetylene chains are in principle energetically more favorable than the *cis*‐chains,^[19]^ but here the different stereoisomers appear to be locally stochastically distributed, which results in a main orientation of the polymeric chains along the average [$1\bar{2}1$ ]‐directions (Figure 2).

**Figure 3:**
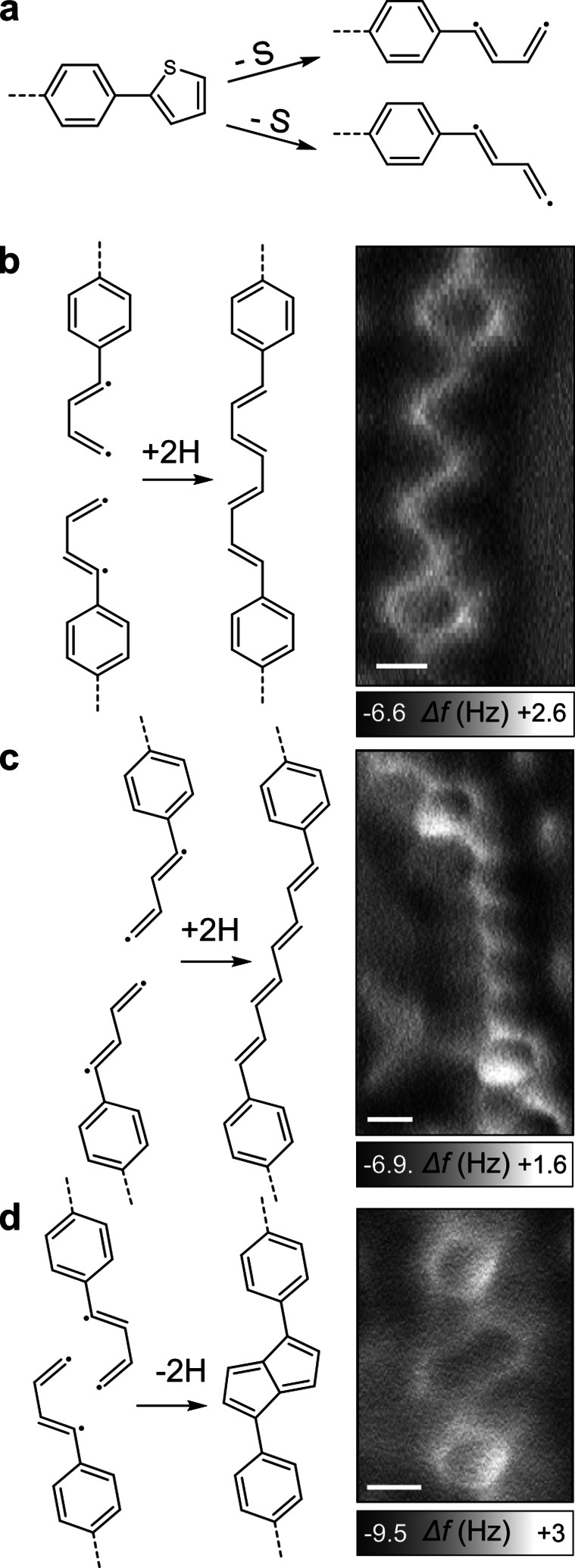
Schematic drawings of the different coupling schemes along with representative high‐resolution AFM images. a) Dissociation of the thiophene rings. Formation of b) *cis*‐oligoacetylene chains, c) *trans*‐oligoacetylene chains and d) pentalene. Scale bars: 2 Å.

Figure 3 shows the different reaction products that are formed locally, starting from the desulfurization of the thiophene units. In principle, the thiophene ring opening reaction creates bi‐radical species, which couple predominantly at one radical site. The resulting polymeric radical chains are subsequently hydrogenated to produce unsaturated closed shell hydrocarbons (see below).

**Figure 4:**
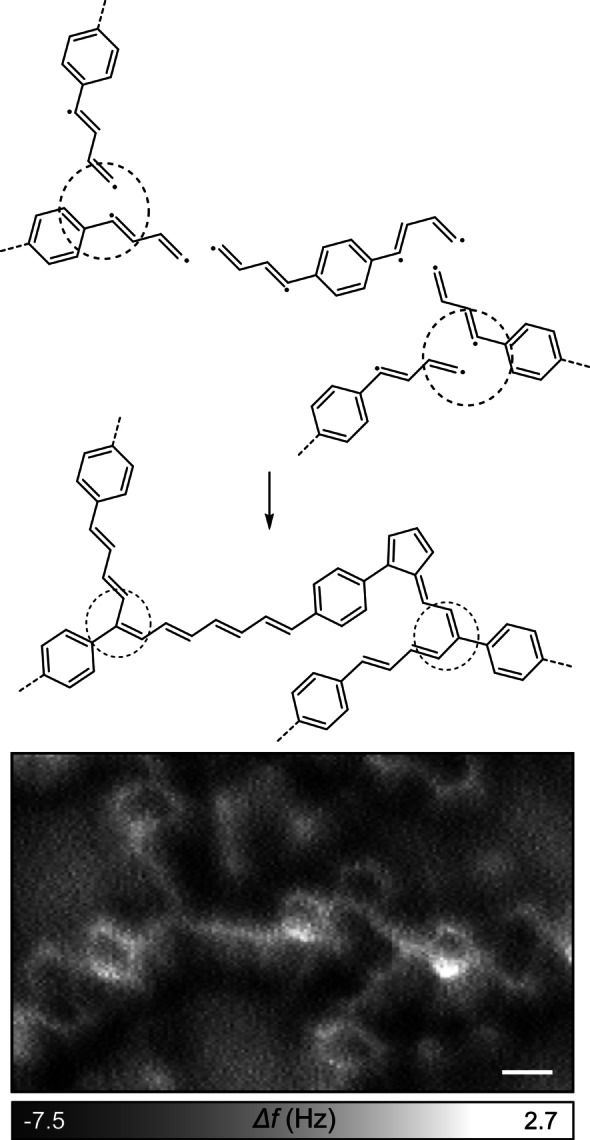
Schematic drawing of the suggested coupling schemes leading to the threefold crossings (dashed circles) along with the corresponding high‐resolution AFM image. Scale bar: 2.5 Å.

Along with *cis*‐ and *trans*‐oligoacetylene chains, pentalene units (Figure 3d) are also formed occasionally, with an occurrence lower than 20 %. The darker contrast measured on these cyclic structures was well reproduced by DFT calculations (Supporting Information Figure S3b). Besides the bifunctional coupling mechanism that creates linear chains, threefold crossing connections are also found. These are responsible for the reticulation of the polymeric network as observed at larger scales (Figure 2a, b). A high‐resolution image of threefold connections is reproduced in Figure 4 along with suggestions of their chemical structure. These connections are obtained by coupling of the terminal carbon to the carbon radical closest to the benzene ring. Similar to the formation of pentalene, the creation of these threefold connections allows for a more advanced coupling reaction of the biradical species.

The hydrogenation of radical products is a common process in on‐surface synthesis for which the H supply may have different origins. The C−C coupling reaction leading to the formation of pentalene (Figure 3d) or chain interconnections (Figure 4) spontaneously releases hydrogen atoms that recombine with the carbon radicals,^[20]^ although probably in understoichiometric ratio. Atomic hydrogen could be further produced at step edges from degraded molecules.^[20a]^ The residual hydrogen gas from the vacuum chamber could represent an additional hydrogen source, as it was similarly suggested for various other systems.^[21]^ The presence of a carbon radical would lead to strong distortions inside the chains (see Supporting Information Figure S4) and to a substantial chemical shift^[22]^ of the C 1s core level that are not compatible with the perfectly flat contrast of the structures observed in AFM images and XPS data. Also, we never observed products in which the C−S bonds are replaced by C−H bonds without C−C coupling, thus suggesting that the hydrogenation takes place as the last reaction step. Interestingly, no C−Cu bond was identified by XPS at this stage (see corresponding text below). While the complexation of radical species with adatoms is usually observed as an intermediate step in Ullmann‐like coupling reactions,[^6a^, ^22^, ^23^] in the present case the C−C coupling followed by hydrogenation takes place without any observable organometallic intermediate.

In addition to the carbon chains, small, isolated dots (see Figure 2c, d) that we can assign as sulfur byproducts are observed in the vicinity of the polymeric chains. They are mostly aligned along the [$1\bar{2}1$ ]‐directions and regularly positioned with an interspacing of 4.3±0.1 Å thus compatible with an epitaxial relationship along this direction (for Cu(111), *a* $\sqrt{3}$ =4.4 Å).

**Figure 5:**
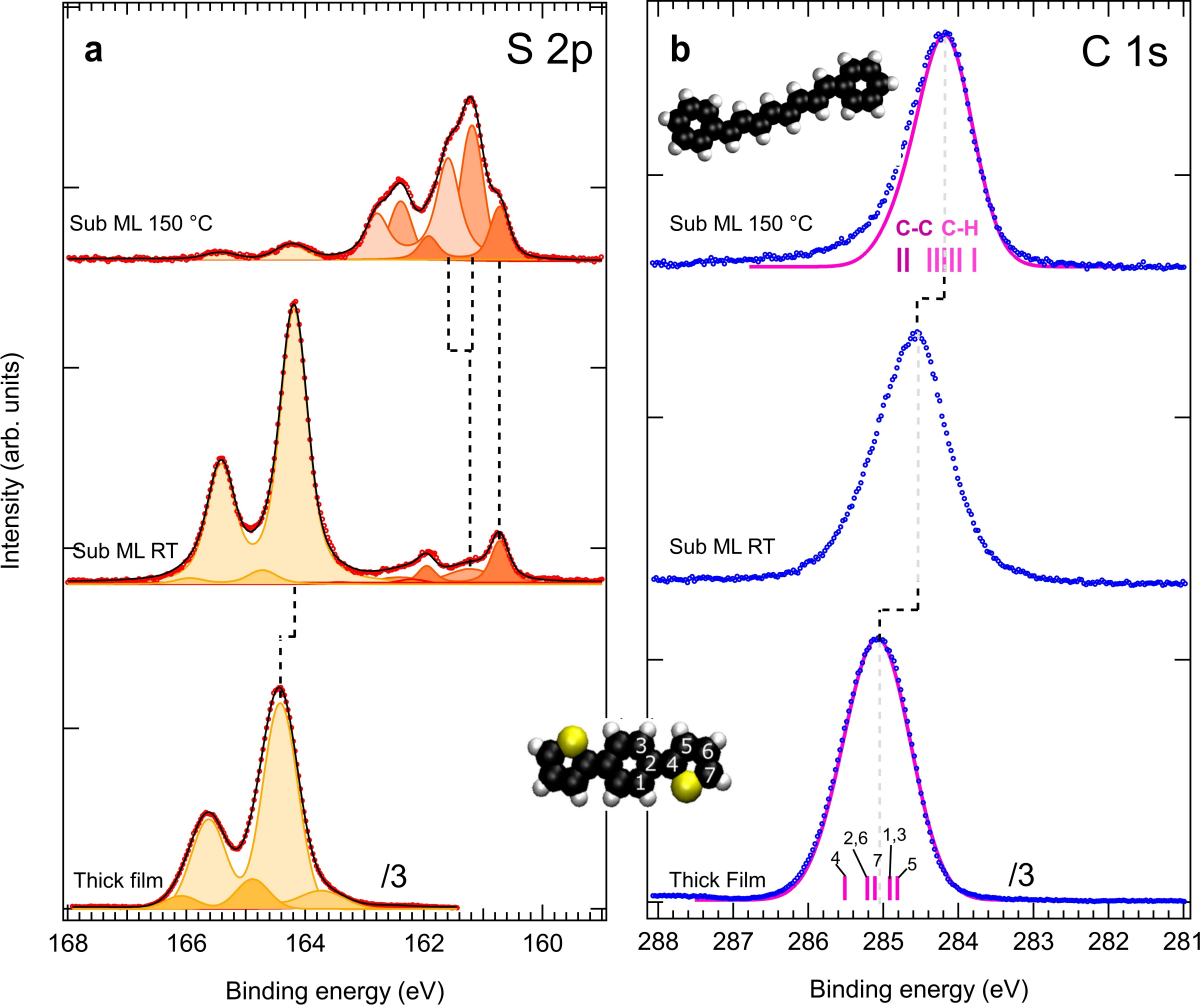
XPS data for DTB/Cu(111). Bottom spectra: a thick film deposited at RT. Upper spectra: sub‐ML coverage deposited at RT and annealed at 150 °C. a) S 2p core level (*hν*=260 eV). The vertical dashed lines are intended to follow the evolution of the different components (peak fitting) as explained in the text. b) C 1s core level (*hν*=382 eV). Markers: experimental data. Magenta full lines: DFT simulated core levels. For the DTB (thick film) the calculated core levels are numbered according to the inset molecular model. For the polyene chain (sub ML 150 °C) the C−C and C−H eigenstates are colored differently.

To gain further insight into the chemistry at play in this system, we acquired temperature‐dependent XPS spectra. The S 2p spectrum (Figure 5a) shows the evolution of the chemical state of S atoms during the polymerization process. The spectrum of a thick layer is used as fingerprint of the pristine molecule. It displays a strong component at a binding energy (BE) of 164.4 eV, in line with other thiophene derivatives on the same surface.[^6a^, ^11a^] Least‐square fitting gives a full width at half maximum (FWHM) of 0.72 eV, essentially due to intermolecular π‐stacking interactions. Two minor components are added to improve the fit which are likely due to different final state screening of surface (high BE) and interface (low BE) molecules.

When a submonolayer is deposited at room temperature, the DTB‐derived peak is narrower (FWHM=0.55 eV), close to what is observed in the gas phase for other thiophene derivatives.^[24]^ This is consistent with the 2D gas‐phase observed by RT‐STM and the consequent reduced intermolecular interaction. The observed shift to lower BE (by −0.24 eV) is characteristic of a reduced film thickness due to the enhanced screening from the metal substrate. An additional minor component at higher BE may be ascribed to a certain preferred adsorption site. At about 3 eV lower BE (between 160.5 and 162.5 eV) a new spectroscopic feature appears revealing atoms having left the molecule upon thiophene ring opening.[^6a^, ^11a^] A possible fragmentation of the thiophene into thiolates (e. g., CHS) adsorbed on Cu(111) should give a BE of the 2p_3/2_ component around 162.0–162.5 eV,^[25]^ which is clearly not observed here. The positions and the line shape rather reveal the presence of atomic S coordinated with Cu.[^25b^, ^26^]

When the temperature is increased to 150 °C, i. e., after polymerization, the spectral weight of DTB transfers almost completely into the low BE feature which now distinctively shows three atomic‐S derived components at 161.6, 161.2 and 160.7 eV (see also Figure S5). The first two components were previously reported for sub‐ML coverage of S/Cu(111)^[26a]^ and can be related to the formation of various S−Cu coordination structures in the vicinity of step edges. In fact, when adsorbed on Cu(111), the high affinity of S to Cu produces mass transport and step edge reconstruction leading to the formation of complex fourfold coordination sites.^[27]^ The step edge regions shown in Supporting Information Figure S6 are clearly active sites for molecular adsorption. On the other hand, the lowest BE component at 160.7 eV was not observed in previous studies on atomic sulfur, thiols or thiolates adsorption on Cu(111).[^6a^, ^11a^, ^25a^, ^25b^, ^26^] We assign this component to the presence of lower‐coordination S, i. e., terrace‐supported, threefold‐coordinated S. This feature can be traced back to the small protrusions observed beside the polymer chains in the AFM images (see Figure 2c,d). Such protrusions are smaller than the usual S−Cu complexes[^27a^, ^27b^] and are likely to be isolated S atoms restricted from diffusing by the interaction with the just‐formed nearby polymers.

The C 1s spectrum (Figure 5b) reflects the presence of C atoms in different oxidation states within the DTB molecule and in the polymeric chains, as well as their interaction with the substrate. When possible, the C 1s spectrum was modeled by DFT calculations. As for the S 2p spectrum, the C 1s of the thick film is representative of the different C atoms in the pristine DTB molecule. The modeling using a DFT‐calculated set of Gaussian components (FWHM of 0.9 eV) represents a good fit for the measured spectrum. The small asymmetry observed at high BE can be attributed to the S‐bonded C atom (labeled 4 in inset Figure 5).

Differently to what is found for S 2p, when a submonolayer of DTB is deposited a visible broadening is observed for C 1s. Additionally, a larger shift of −0.48 eV towards lower BE is observed. This suggests that the molecule‐substrate interaction occurs through C atoms (as compared with S atoms) that are found in a number of inequivalent sites with well‐screened final states. Part of the broadening at the low‐BE side can also be ascribed to the presence of a small fraction of molecules having started to react and lost their S atoms, as revealed by the corresponding S 2p spectrum.

Annealing the sub‐ML from RT to 150 °C induces a progressive shift to low BE concurrent with the thiophene ring opening and S release (see Supporting Information Figure S5). At the same time the spectrum narrows, testifying to a more homogeneous sample with well‐defined atomic sites within the polymer chains. Its BE and line shape are very similar to those of other 2D polymer‐derived spectra.[^19^, ^22^, ^28^] A simple modeling was performed by considering octatetraene chains terminated by two phenyl units adsorbed on the surface (Supporting Information Figure S3a). This model reproduces the overall line shape with a pronounced asymmetry to high BE due to the presence of minority C's having three C−C bonds (the phenyl C's linking the oligoacetylene chains). In the simulated spectra, the inclusion of the Cu(111) surface does not affect the line shape (see Figure S7). Compared to the simulated model, the real system includes more complex conformations (pentalene structure and threefold connection branches) with a larger C−C to C−H ratio that could be at the origin of the discrepancy in the high BE region. The C−C to C−H BE difference was previously detected in other on‐surface polymerization studies involving aromatic compounds,[^22^, ^28a^, ^28c^, ^28d^, ^29^] although sometimes with opposite shifts.[^28b^, ^30^]

The XPS data shows that the loss of carbon upon annealing is limited to ∼3 %, in similar proportion as the loss of S (∼2 %), so we can conclude that most of the carbon and sulfur atoms remain on the surface. The yield of the C−S activated reaction is thus very high and takes place in the range 50 to 120 °C (Figure S5).

**Figure 6:**
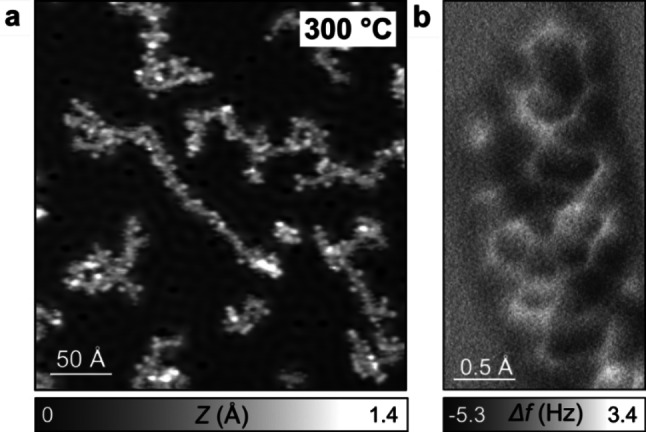
a) STM and b) AFM images of DTB/Cu(111) after annealing at 300 °C.

Figure 6 shows representative STM and AFM images of the system obtained after annealing at 300 °C. We could not observe a transition to the more favorable *trans*‐chain configuration,^[19]^ probably due to the strongly reduced mobility in this reticulated network. Similar to the case of polyacetylene chains,^[19]^ cyclization reactions take place and lead to the formation of narrow polyaromatic ribbons. Upon further annealing to 500 °C the graphitization reaction is advanced further and small graphene flakes are formed on the surface (see Supporting Information Figures S8, S9). Similar amorphous phases obtained from massive dehydrogenative coupling have been observed for various systems after high temperature annealing of covalent networks on noble metal surfaces.[^23b^, ^31^] Indeed graphene formation can take place on a copper surface at sufficiently high temperature from virtually any carbon source.^[32]^

## Conclusions and Perspectives

We presented the formation of a fully conjugated hydrocarbon polymeric network from simple thiophene‐functionalized precursors. The C−S activation at 120 °C annealing temperature initiates the formation of carbon radicals that couple to form oligoacetylene chains or pentalene units linking the original benzene rings. Although the coupling reaction is not selective towards *cis*‐ or *trans*‐stereoisomers, the mainly linear chains maintain an overall [1$\bar{2}1$ ]‐orientation along the high symmetry directions of the Cu(111) surface. An alternate reaction mechanism occasionally takes place that leads to the creation of threefold interconnections and the overall formation of a reticulated network. Upon further annealing, additional cyclodehydrogenation reactions occur, leading finally to the formation of small graphene flakes up to a temperature of 500 °C.

Our study demonstrates that the strong reactivity of copper toward sulfur‐containing species can be exploited to steer an on‐surface homocoupling reaction and to create extended polyene chains at low activation temperature. Unfused thiophene precursors offer simpler synthesis and more diverse coupling chemistry than fused precursors,^[12b]^ as they form diradical butadiene moieties instead of ethylene monoradicals. For the synthetic approach in general, C−S activation could be complementary to Ullmann‐type coupling as it provides access to antiaromatic and nonbenzenoid structures, such as the pentalene minor product we obtained. However, the precise reaction mechanism still needs to be elucidated, in particular with regard to the role of the atomic sulfur, that is known to affect the bonding of hydrocarbons on Cu(111).^[33]^ Compared to Ullmann‐like coupling of halogenated molecules,[^6a^, ^22^, ^23^] no organometallic intermediate was detected, which suggests that the desulfurization step is rate‐limiting with respect to both the C−C coupling and the hydrogenation reactions. This is also consistent with the similarly low activation temperatures that were reported previously for alkenyl C−C coupling.[^19^, ^23a^] In future work, different strategies^[1e]^ may be explored to improve the order and density of the polymeric network. Preliminary experiments on Cu(110) (see Figure S10) showed that the strong anisotropy of this surface did not noticeably steer the alignment of the chains, but alternate solutions like for example the use of vicinal surfaces,^[34]^ co‐adsorbed oxygen^[35]^ or hydrogen,^[36]^ or supramolecular templating^[37]^ may still deliver interesting results.

Thiophene groups are sometimes used as constitutive groups in on‐surface synthesis on various metal surfaces.[^6a^, ^6c^, ^7^, ^8^] In view of our results showing a relatively low activation temperature of the thiophene ring opening reaction, it is certainly necessary to confirm the integrity of these monomers. Conversely, solutions to preserve the integrity of sulfur in on‐surface polymerization reactions include providing an out‐of‐plane structure of the precursors like in diamantanethiol,^[38]^ or following other strategies that have proved successful to maintain specific functions even on reactive surfaces.^[39]^

## Conflict of interest

The authors declare no conflict of interest.

1

## Supporting information

As a service to our authors and readers, this journal provides supporting information supplied by the authors. Such materials are peer reviewed and may be re‐organized for online delivery, but are not copy‐edited or typeset. Technical support issues arising from supporting information (other than missing files) should be addressed to the authors.

Supporting Information

## Data Availability

The data that support the findings of this study are available from the corresponding author upon reasonable request.
