# Supplementary material for: On‐Surface Synthesis of Unsaturated Hydrocarbon Chains through C−S Activation
Source: Chemistry. 2022 Jul 11;28(47):e202200809. doi: 10.1002/chem.202200809 (PMC9540368; doi:10.1002/chem.202200809)
Supplement: Supplementary file 1 — Supporting Information [file CHEM-28-0-s001.pdf]

Chemistry–A European Journal

Supporting Information

On-Surface Synthesis of Unsaturated Hydrocarbon Chains through C–S Activation

Luca Giovanelli,* Rémy Pawlak, Fatima Hussein, Oliver MacLean, Federico Rosei, Wentao Song, Corentin Pigot, Frédéric Dumur, Didier Gigmes, Younal Ksari, Federica Bondino, Elena Magnano, Ernst Meyer, and Sylvain Clair*

Methods

Chemical synthesis of 1,4-di(thiophen-2-yl)benzene

Tetrakis(triphenylphosphine)palladium (0) (0.46 g, 0.744 mmol, M = 1155.56 $g.mol^{-1}$) was added to a mixture of 1,4-dibromobenzene (1.44 g, 6.11 mmol, M = 235.90 $g.mol^{-1}$), 2-thiopheneboronic acid (1.62 g, 12.66 mmol, M = 127.96 $g.mol^{-1}$), toluene (54 mL), ethanol 26 mL) and an aqueous potassium carbonate solution (2 M, 6.91 g in 25 mL water, 26 mL) under vigorous stirring. The mixture was stirred at 80 °C for 48 h under a nitrogen atmosphere. After cooling to room temperature, the reaction mixture was poured into water and extracted with ethyl acetate. The organic layer was washed with brine several times, and the solvent was then evaporated. Addition of DCM followed by pentane precipitated a white solid which was filtered off. The residue was purified by column chromatography (SiO2, pentane/DCM: 1/1 and pure DCM) and isolated as a white solid (1.15 g, 78% yield). ^{1}H NMR, see Figure S11 (250 MHz, $CDCl_3$) δ 7.63 (s, 4H), 7.32 (dd, *J* = 11.9, 3.9 Hz, 4H), 7.14 – 7.05 (m, 2H); ^{13}C NMR, see Figure S12 (63 MHz, $CDCl_3$) δ 143.87, 140.28, 133.45, 128.08, 126.27, 124.88, 123.07, 77.50, 76.99, 76.49; HRMS (ESI MS) m/z: theor: 242.0224 found: 242.0224 (M^+ detected).

Scanning Probe Microscopy

The Cu(111) single crystal was cleaned by repeated cycles of Ar sputtering and annealing. The DTB molecules were deposited from a Knudsen cell maintained at a temperature of 60 to 70°C. The experiments were performed in two different setups. Room-temperature STM was performed with an Omicron VT-STM (typical tunneling parameters 300 pA, -0.5 ~ -1.5V). Low-temperature STM/AFM images were acquired at T = 4.8 K in ultrahigh vacuum ($p \approx 1\times10^{-10}$ mbar) using an Omicron microscope operated with a Nanonis RC5 electronic controller. The force sensor is a commercially available tuning fork sensor based on a qPlus design operated in the frequency-modulation mode (resonance frequency $f_0 \approx 25$ kHz, spring constant $k \approx 1800$ $N.m^{-1}$, quality factor Q = 14000 and oscillation amplitude A ≈ 0.5 Å). STM images were acquired in constant-current mode with the voltage applied to the tip. AFM measurements were acquired in constant-height mode at V = 0 V. The tip mounted to the qPlus sensor consists in a 25 µm-thick PtIr wire sharpened *ex situ* using a focused ion beam. A clean and sharp Cu tip was then prepared at low temperature by repeated indentations into the substrate. A functionalized tip was created by picking up a single CO molecule from the surface.[99] The images were partly treated with the free software WSxM[100] and Gwyddion.[101]

Photoemission Spectroscopy

XPS was performed *in-situ* with synchrotron radiation at the IOM-CNR BACH beamline (Elettra, Trieste). DTB was evaporated in UHV on the substrate kept at RT. After molecular deposition, the sample was annealed in the analysis chamber (base pressure $1x10^{-9}$ mbar) through a filament at the back of the sample. XPS was performed with a Scienta 3000 electron energy analyzer, in normal emission and using horizontally polarized light. XPS peak fitting of the Shirley background-subtracted S 2p spectra was performed by using Voigt peaks with fixed Lorentzian width of 0.3 eV. The spin-orbit splitting was fixed to the usual 1.2 eV value[67] while the branching ratio was adjusted around the multiplicity value (2:1).

Theoretical modeling

Theoretical calculations were performed with the Vienna *Ab initio* Simulation Package (VASP)[102,103] using the Perdew-Burke-Ernzerhof[104] generalized-gradient approximation (PBE-GGA) for the exchange-correlation potential, the projector augmented wave (PAW) method,[105,106] and a plane-wave cutoff of 450 eV. The zero-damping DFT-D3 method of Grimme[107] was used for the van der Waals (vdW) correction of the potential energy, and calculations were performed at the gamma *k*-point. Geometry optimizations for the AFM simulations were performed with an (8×8) orthogonal Cu(111) slab constructed using a lattice constant of 0.363 nm and a 1.8 nm vacuum layer, with four atomic layers and the positions of atoms in the bottom two layers fixed (supercell dimensions: 2.05620 nm × 17.8073 nm × 24.2958 nm). Calculations for DTB on Cu(111) used a smaller (6×6) orthogonal slab with the same lattice constant, five atomic layers (bottom two fixed), and a 1.6 nm vacuum layer. The geometries were optimized until the force on each atom was below 0.02 eV/Å. Images of the calculated structures were generated using the VMD software.[108] The AFM image simulation was performed using the Probe Particle model[109,110] for a CO-terminated tip with a charge of 0.05 e, lateral bending stiffness of 0.5 N/m and a particle-tip bond stiffness of 20.0 N/m. The simulated XPS spectra are the result of the convolution of several Gaussians centered at the calculated core level positions (vertical ticks). A negative global shift was applied to the calculated energies to match the maxima of the experimental spectra.

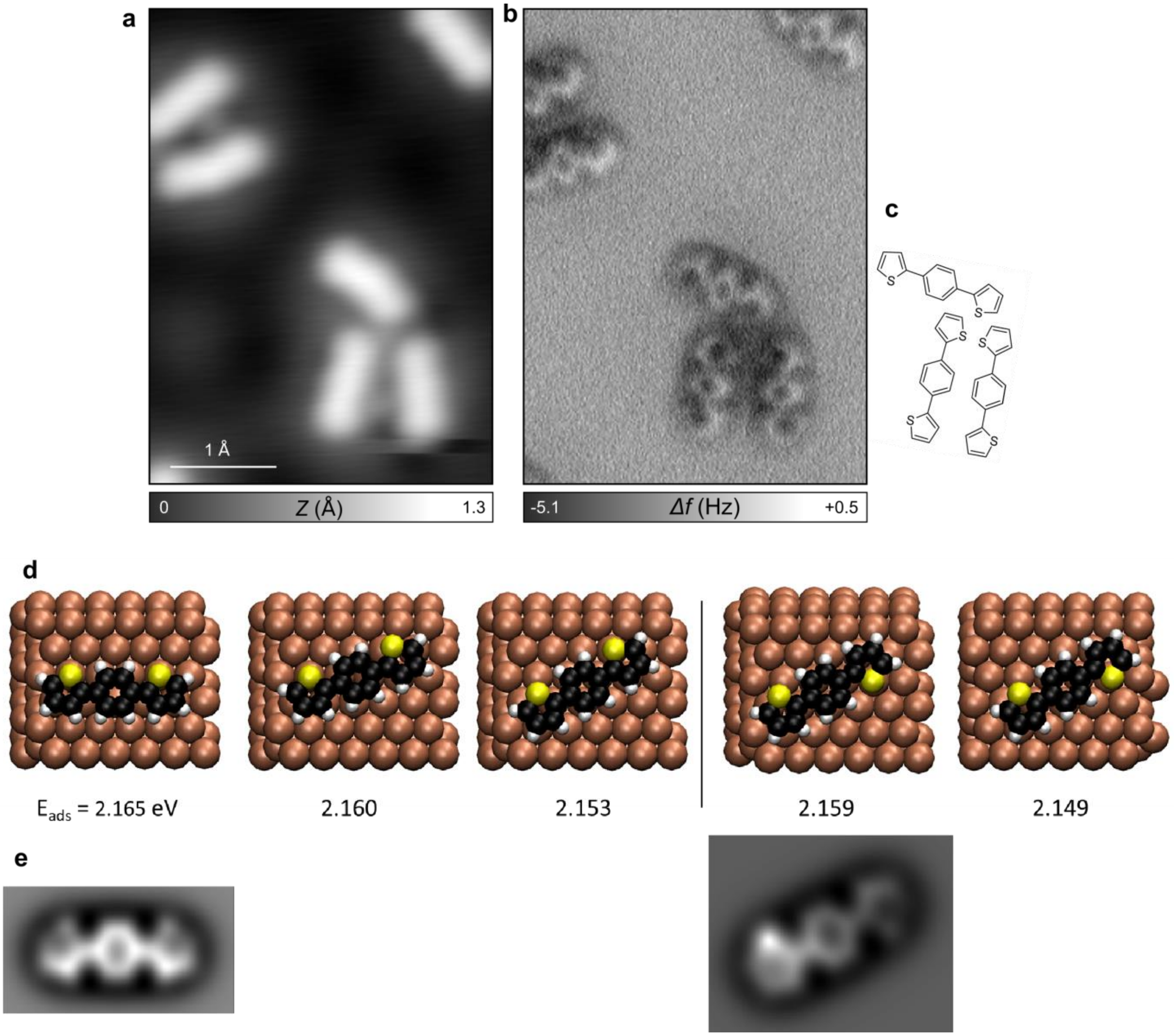

Figure S1. STM (a), corresponding AFM image with CO-terminated tip (b) and schematic model (c) of isolated DTB molecules adsorbed with different orientations of the *syn*- and *anti*-conformers. (d) Relaxed structures obtained from DFT calculations and (e) corresponding AFM image simulations. The molecular adsorption energies vary by less than 20 meV for the different configurations.

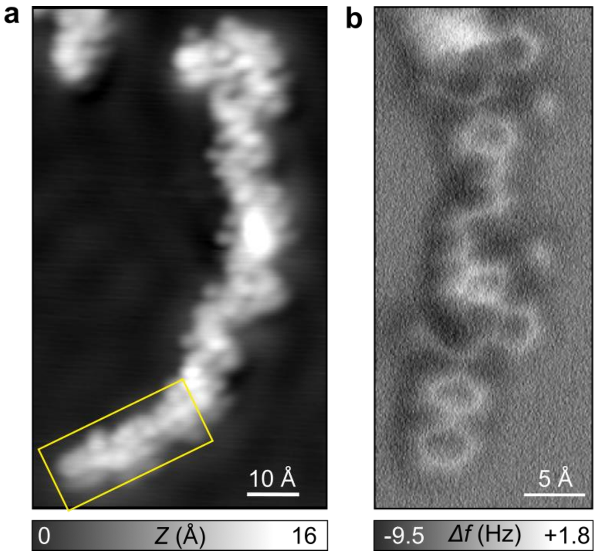

Figure S2. (a) STM image of the polymeric chains obtained after annealing the Cu(111) sample between 80-100°C. (b) AFM image acquired in the yellow rectangle highlighted in (a) with a CO-terminated tip. The chains are less ordered and unreacted thiophene end-groups are still present.

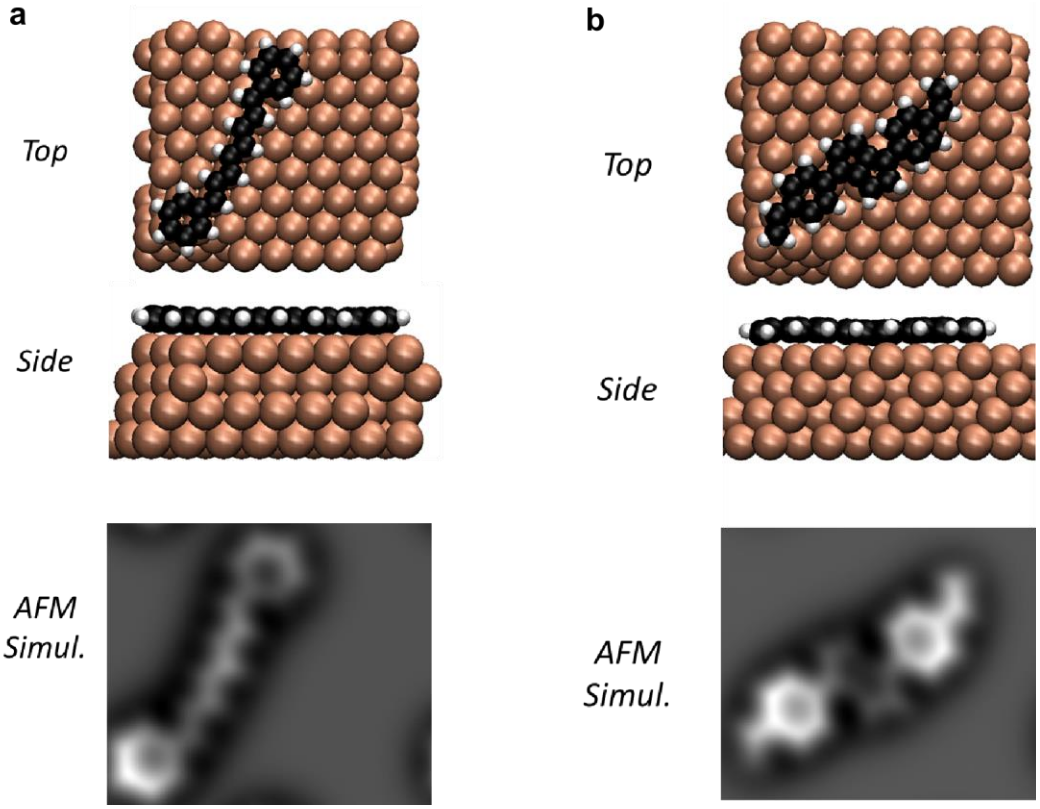

Figure S3. DFT Simulations. (a) Atomic structure of a relaxed diphenyl-*trans*-octatetraene model compound on Cu(111) showing its orientation along the [$1\bar{1}0$]-direction and corresponding AFM image simulation. (b) Atomic structure of a relaxed diphenylpentalene and corresponding AFM image simulation.

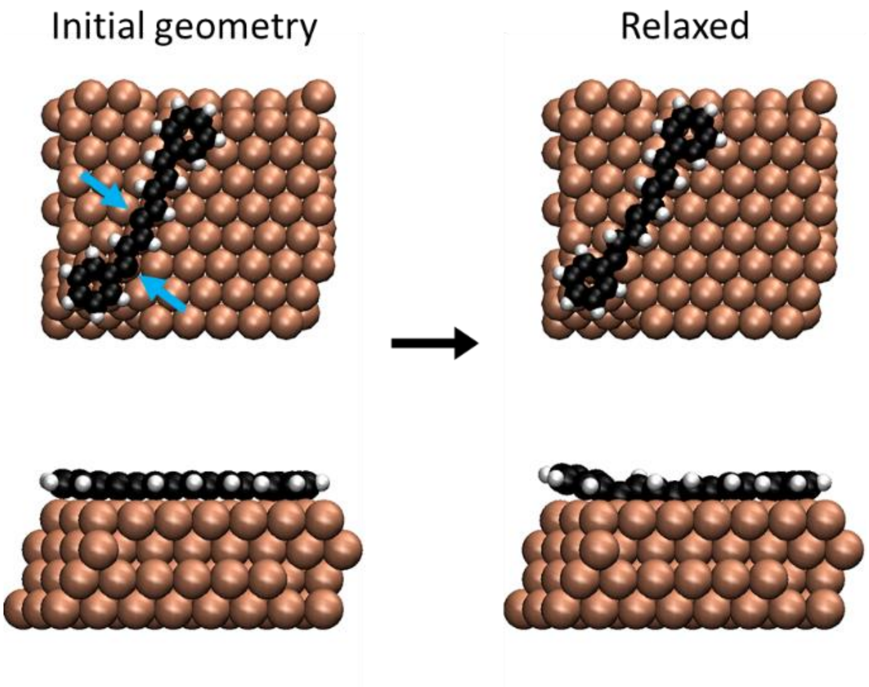

Fig S4. DFT simulations showing the relaxation of the radical sites (blue arrows) in diphenyl-*trans*-octatetraene and the induced twist, incompatible with the perfectly flat contrast of all structures observed experimentally. Free radicals have a natural tendency to sit in upright position on the copper surface.[1,2] The chemical shift of the C radical with respect to C-H was estimated to be -1.1 eV toward low BE. This is not compatible with the measured spectral shape of Figures 5 and S4, giving further evidence that the chains have been hydrogenated.

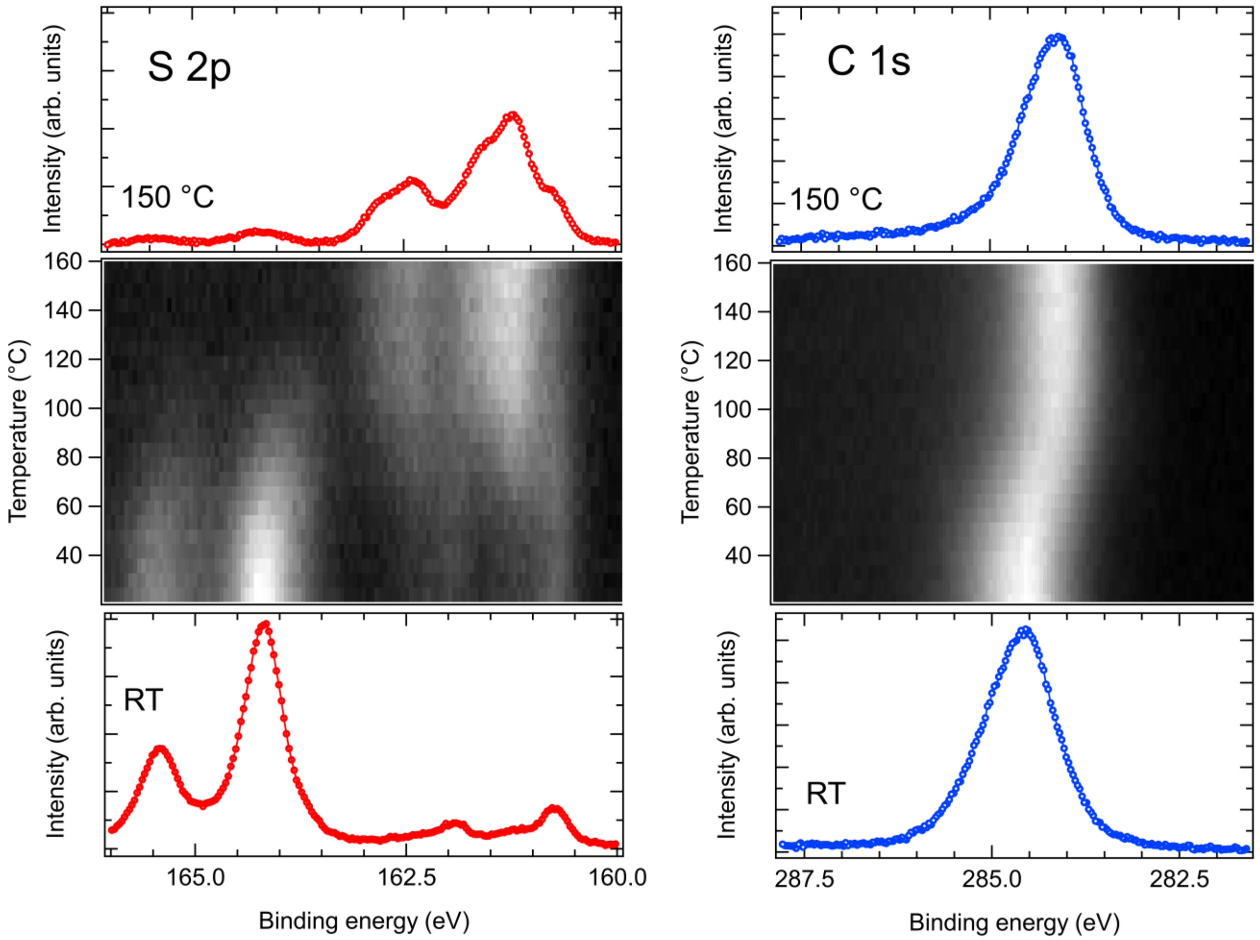

Figure S5. S 2p and C 1s temperature-programmed XPS (central panels) of a sub-ML DTB/Cu(111) from RT to 150 °C (annealing rate was 2.4 °C/min). Below and above are the high resolution spectra at initial and final temperatures. Starting from about 40 °C, the thiophene S 2p component clearly transforms into the three atomic sulfur doublets. At the same time, the C 1s gradually shifts to attain the BE corresponding to the polymeric chains. The reaction is completed at around 120 °C.

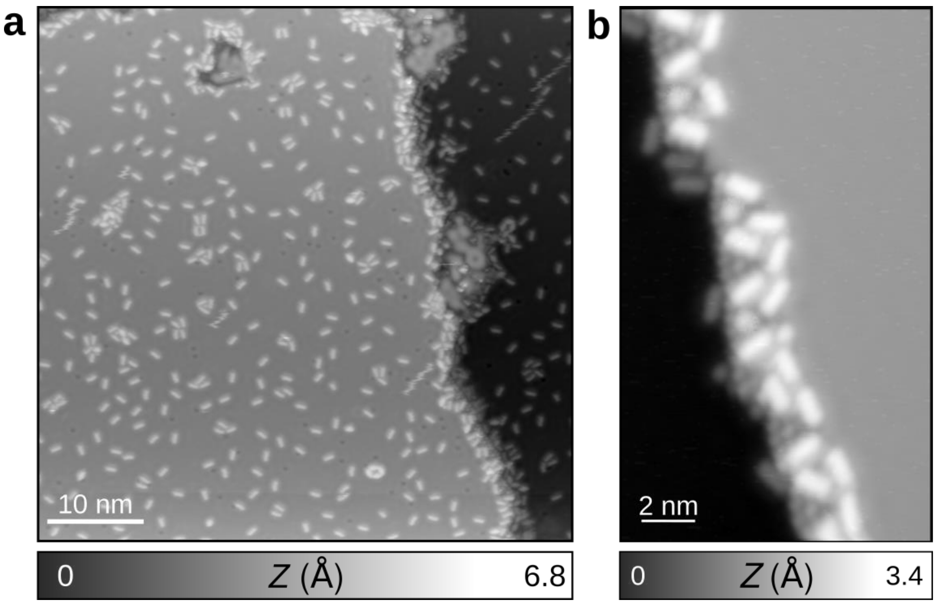

Fig S6. STM topographic images acquired at 4.8 K after sublimation on the Cu(111) sample kept at RT (a) showing step edge decoration (b).

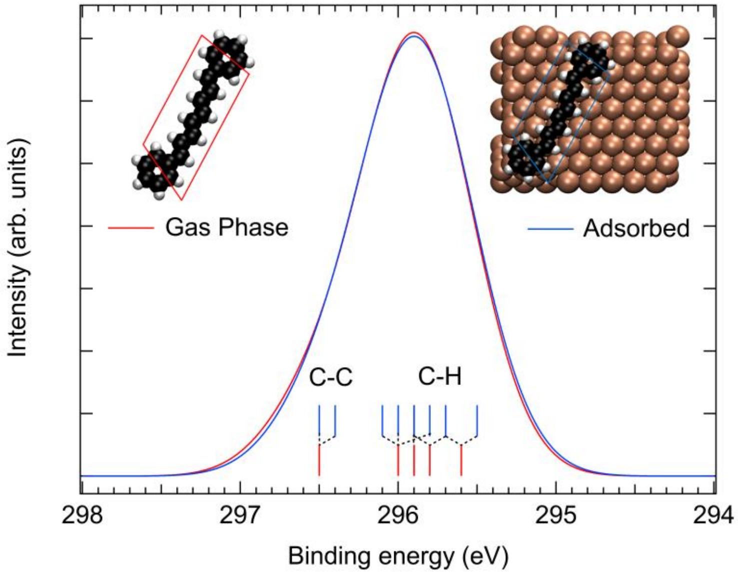

Figure S7. C 1s calculated spectra for the diphenyl-octatetraene molecule in gas phase and adsorbed on Cu(111). The spectra result from convolution of Gaussian curves (FWHM= 0.75 eV) centered at the calculated C 1s BE (bars at the bottom). The evolution of the atomic core level BE is indicated at the bottom. No significant difference in the overall spectral shape can be observed between the two models.

Morphology and electronic structure after annealing at high temperature

As stated in the manuscript, when the annealing temperature is raised above 300 °C, the cyclization reactions proceeds towards graphitization in larger domains. This is seen in Figure S7 where SPM images of the cyclization (300 °C) and graphitization (500 °C) are displayed.

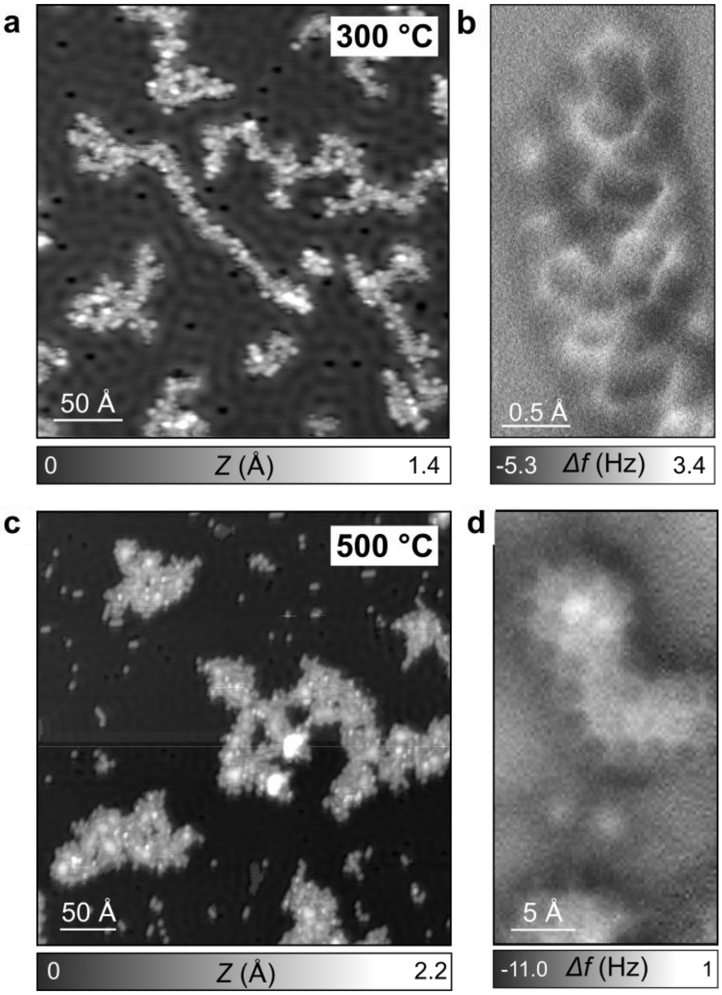

Figure S8. STM (a,c) and AFM images (b,d) of DTB/Cu(111) after annealing at 300°C (a,b) and 500°C (c,d). The images (a,b) are identical to those presented in Figure6 of the main text.

The cyclization reaction process is visible also in the C 1s spectra (Figure S8). Up to 200 °C the spectrum is unchanged with respect to the linear polymer but, from 225 °C onwards, a gradual narrowing occurs, indicating an overall rearrangement towards a more homogeneous C site population. Above 300 °C, the narrowing is accompanied by the emergence and growth of a typical low BE component at 283.2 eV, previously found also in nanographene patches[6,7] and coming from defective or Cu-coordinated C atoms at the edge of polycyclic ribbons and flakes.[8-12] This low BE component does not appear at 150 °C, thus

excluding the possibility of having free or Cu-bonded C radicals in the polymeric chains (see Figures 5 and S4).

Significant changes are observed at high temperature also in the S 2p spectra. The interaction between isolated atomic S and the chains gradually decreases as seen by reduction of the low BE S 2p component (Figure S8). At 325 °C it has completely disappeared to the benefit of the two high-coordination components that eventually merge into a single component at 161.45 eV, representative of copper sulfides formed at high temperatures.[3-5]

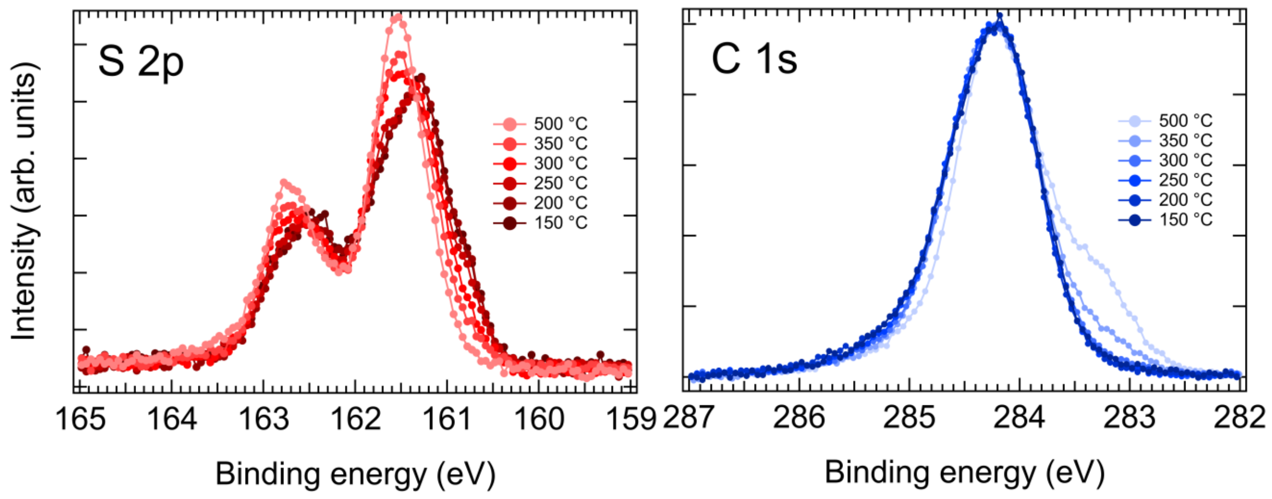

Figure S9. S 2p and C 1s core levels of the polymerized DTB as a function of increasing annealing temperature from 150 °C to 500 °C. The sample was annealed at the indicated temperatures for two minutes and then cooled down before measuring. Left panel (S 2p): above 200 °C a gradual decrease of the low BE component (S adatoms bound to the linear polymer) to the benefit of the two high coordination components is observed. At higher temperatures these components gradually merge into a single copper sulfide peak. Right panel (C 1s, normalized to peak maxima): the asymmetry at high BE reduces gradually above 200 °C (see intensity reduction between 285 and 286 eV) and a C-Cu component emerges at low BE above 300 °C annealing temperatures. To facilitate the comparison, the spectra of samples annealed at 350 °C and 500 °C were shifted to higher BE (by 0.03 and 0.08 eV, respectively) to match the BE of the peak maxima at 150 °C.

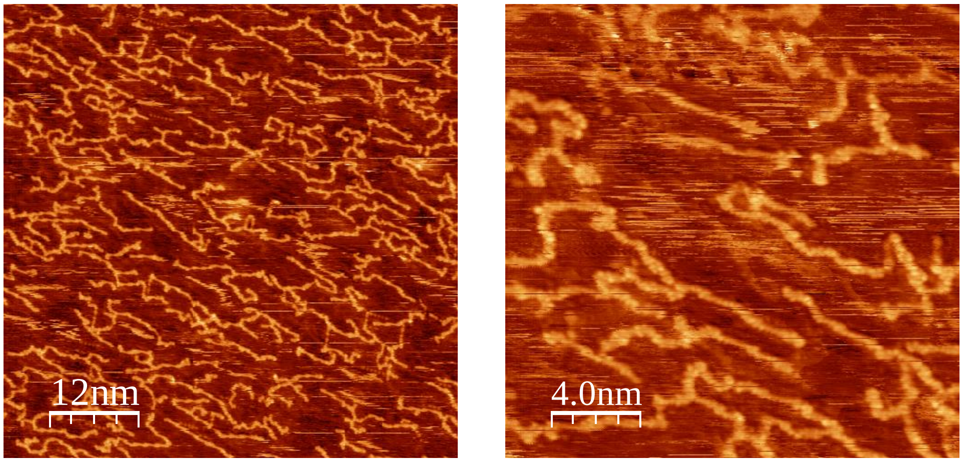

Figure S10. RT-STM images showing the polymer formed on the Cu(110) surface after annealing at 200°C. The alignment of the chains is not particularly improved as compared to the Cu(111) case.

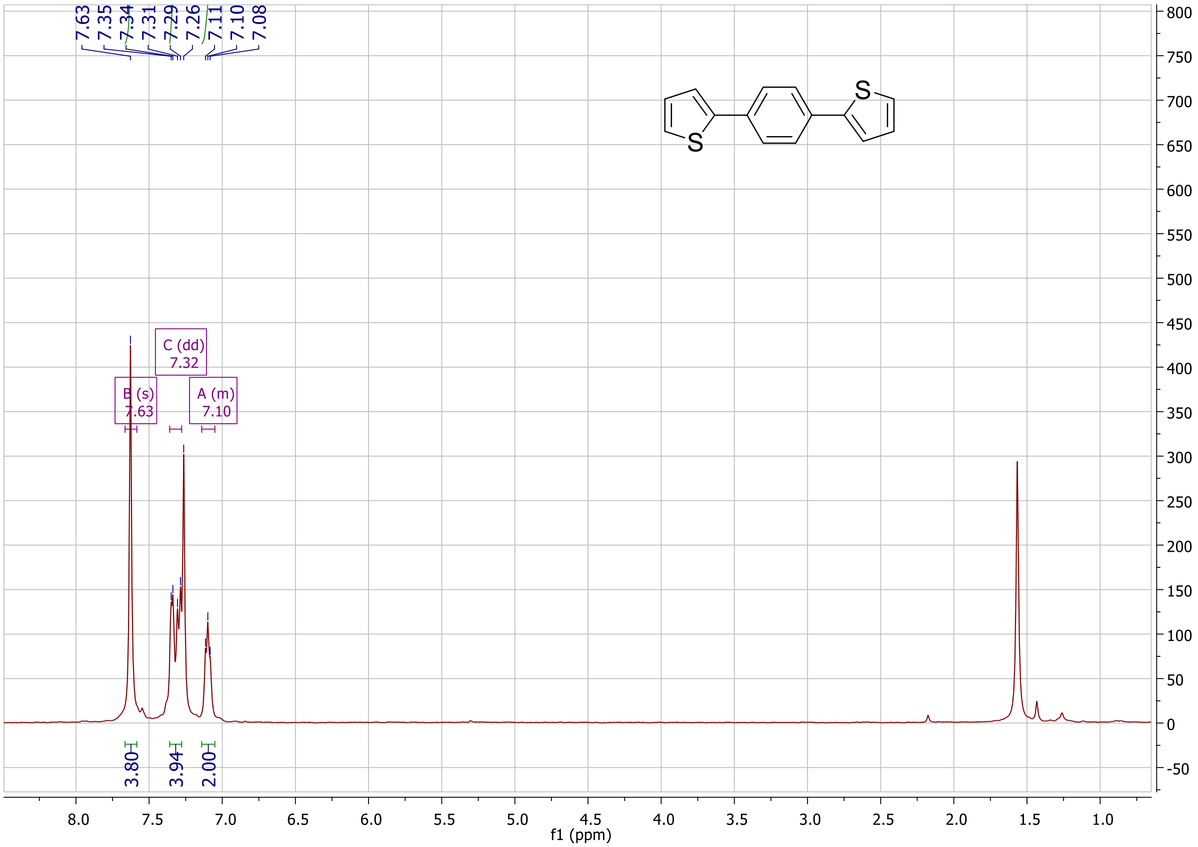

Figure S11. ^{1}H NMR spectrum of 1,4-di(thiophen-2-yl)benzene (DTB)

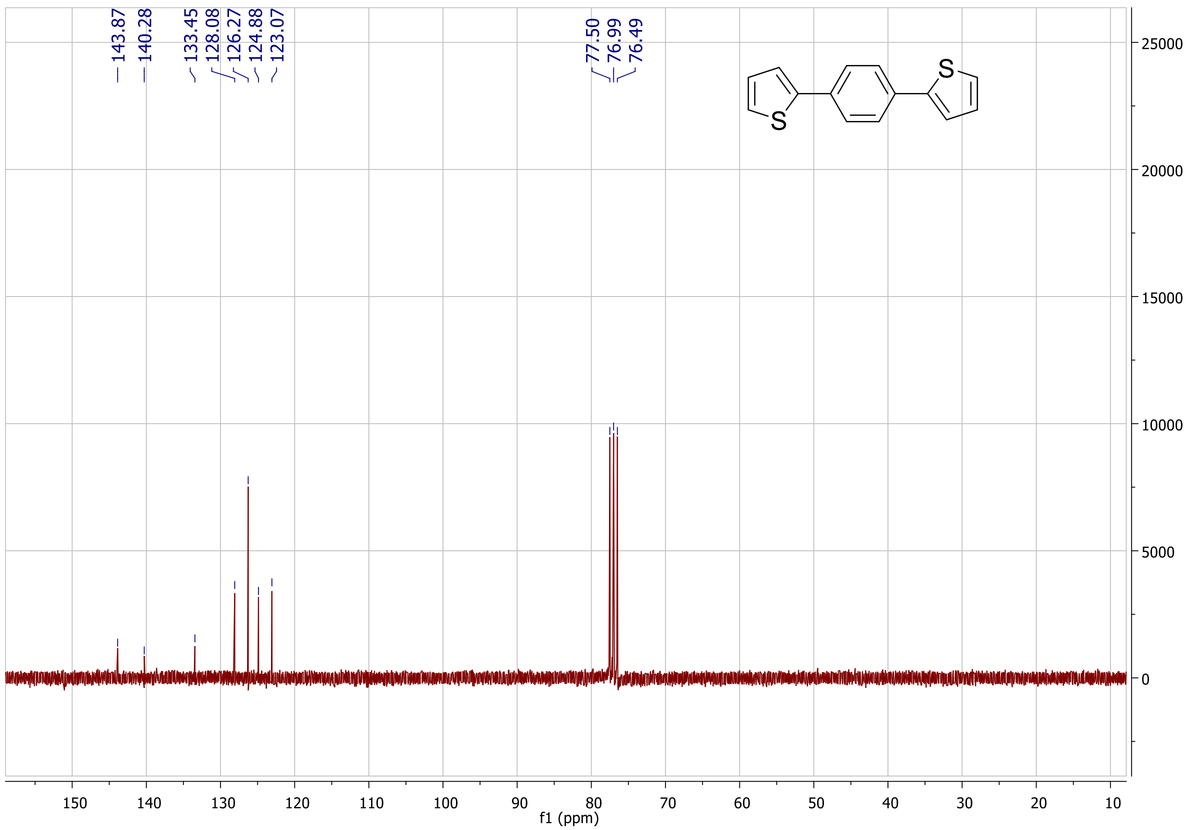

Figure S12. ^{13}C NMR spectrum of 1,4-di(thiophen-2-yl)benzene (DTB).

References

(1) Björk, J. Reaction Mechanisms for On-Surface Synthesis of Covalent Nanostructures. *J. Phys. Condens. Matter.* **2016,** *28* (8), 083002.

(2) Du, Q. Y.; Pu, W. W.; Sun, Z. R.; Yu, P. On-Surface Synthesis of All-cis Standing Phenanthrene Polymers upon Selective C-H Bond Activation. *J. Phys. Chem. Lett.* **2020,** *11* (13), 5022.

(3) Sirtl, T.; Lischka, M.; Eichhorn, J.; Rastgoo-Lahrood, A.; Strunskus, T.; Heckl, W. M.; Lackinger, M. From Benzenetrithiolate Self-Assembly to Copper Sulfide Adlayers on Cu(111): Temperature-Induced Irreversible and Reversible Phase Transitions. *J. Phys. Chem. C* **2014,** *118* (7), 3590.

(4) Di Bernardo, I.; Hines, P.; Abyazisani, M.; Motta, N.; MacLeod, J.; Lipton-Duffin, J. On-Surface Synthesis of Polyethylenedioxythiophene. *Chem. Commun.* **2018,** *54* (30), 3723.

(5) Jia, J. J.; Bendounan, A.; Chaouchi, K.; Esaulov, V. A. Sulfur Interaction with Cu(100) and Cu(111) Surfaces: A Photoemission Study. *J. Phys. Chem. C* **2014,** *118* (42), 24583.

(6) Ferrighi, L.; Pis, I.; Nguyen, T. H.; Cattelan, M.; Nappini, S.; Basagni, A.; Parravicini, M.; Papagni, A.; Sedona, F.; Magnano, E.et al. Control of the Intermolecular Coupling of Dibromotetracene on Cu(110) by the Sequential Activation of C-Br and C-H Bonds. *Chem. Eur. J.* **2015,** *21* (15), 5826.

(7) Simonov, K. A.; Vinogradov, N. A.; Vinogradov, A. S.; Generalov, A. V.; Zagrebina, E. M.; Martensson, N.; Cafolla, A. A.; Carpy, T.; Cunniffe, J. P.; Preobrajenski, A. B. Effect of Substrate Chemistry on the Bottom-Up Fabrication of Graphene Nanoribbons: Combined Core-Level Spectroscopy and STM Study. *J. Phys. Chem. C* **2014,** *118* (23), 12532.

(8) Galeotti, G.; Di Giovannantonio, M.; Cupo, A.; Xing, S.; Lipton-Duffin, J.; Ebrahimi, M.; Vasseur, G.; Kierren, B.; Fagot-Revurat, Y.; Tristant, D.et al. An Unexpected Organometallic Intermediate in Surface-Confined Ullmann Coupling. *Nanoscale* **2019,** *11* (16), 7682.

(9) Di Giovannantonio, M.; El Garah, M.; Lipton-Duffin, J.; Meunier, V.; Cardenas, L.; Fagot-Revurat, Y.; Cossaro, A.; Verdini, A.; Perepichka, D. F.; Rosei, F.et al. Insight into Organometallic Intermediate and Its Evolution to Covalent Bonding in Surface-Confined Ullmann Polymerization. *ACS Nano* **2013,** *7* (9), 8190.

(10) Bushell, J.; Carley, A. F.; Coughlin, M.; Davies, P. R.; Edwards, D.; Morgan, D. J.; Parsons, M. The reactive chemisorption of alkyl iodides at Cu(110) and Ag(111) surfaces: A combined STM and XPS study. *J. Phys. Chem. B* **2005,** *109* (19), 9556.
(11) Gutzler, R.; Cardenas, L.; Lipton-Duffin, J.; El Garah, M.; Dinca, L. E.; Szakacs, C. E.; Fu, C. Y.; Gallagher, M.; Vondracek, M.; Rybachuk, M.et al. Ullmann-Type Coupling of Brominated Tetrathienoanthracene on Copper and Silver. *Nanoscale* **2014,** *6* (5), 2660.
(12) Galeotti, G.; Di Giovannantonio, M.; Lipton-Duffin, J.; Ebrahimi, M.; Tebi, S.; Verdini, A.; Floreano, L.; Fagot-Revurat, Y.; Perepichka, D. F.; Rosei, F.et al. The Role of Halogens in On-Surface Ullmann Polymerization. *Faraday Discuss.* **2017,** *204*, 453.
